# The Braincase of the Basal Sauropod Dinosaur *Spinophorosaurus* and 3D Reconstructions of the Cranial Endocast and Inner Ear

**DOI:** 10.1371/journal.pone.0030060

**Published:** 2012-01-17

**Authors:** Fabien Knoll, Lawrence M. Witmer, Francisco Ortega, Ryan C. Ridgely, Daniela Schwarz-Wings

**Affiliations:** 1 Departamento de Paleobiología, Museo Nacional de Ciencias Naturales-CSIC, Madrid, Spain; 2 Department of Biomedical Sciences, Heritage College of Osteopathic Medicine, Ohio University, Athens, Ohio, United States of America; 3 Facultad de Ciencias, Universidad Nacional de Educación a Distancia, Madrid, Spain; 4 Museum für Naturkunde, Leibniz Institute for Research on Evolution and Biodiversity, Humboldt University, Berlin, Germany; Raymond M. Alf Museum of Paleontology, United States of America

## Abstract

**Background:**

Sauropod dinosaurs were the largest animals ever to walk on land, and, as a result, the evolution of their remarkable adaptations has been of great interest. The braincase is of particular interest because it houses the brain and inner ear. However, only a few studies of these structures in sauropods are available to date. Because of the phylogenetic position of *Spinophorosaurus nigerensis* as a basal eusauropod, the braincase has the potential to provide key evidence on the evolutionary transition relative to other dinosaurs.

**Methodology/Principal Findings:**

The only known braincase of *Spinophorosaurus* (‘Argiles de l'Irhazer’, Irhazer Group; Agadez region, Niger) differs significantly from those of the Jurassic sauropods examined, except potentially for *Atlasaurus imelakei* (Tilougguit Formation, Morocco). The basisphenoids of *Spinophorosaurus* and *Atlasaurus* bear basipterygoid processes that are comparable in being directed strongly caudally. The *Spinophorosaurus* specimen was CT scanned, and 3D renderings of the cranial endocast and inner-ear system were generated. The endocast resembles that of most other sauropods in having well-marked pontine and cerebral flexures, a large and oblong pituitary fossa, and in having the brain structure obscured by the former existence of relatively thick meninges and dural venous sinuses. The labyrinth is characterized by long and proportionally slender semicircular canals. This condition recalls, in particular, that of the basal non-sauropod sauropodomorph *Massospondylus* and the basal titanosauriform *Giraffatitan*.

**Conclusions/Significance:**

*Spinophorosaurus* has a moderately derived paleoneuroanatomical pattern. In contrast to what might be expected early within a lineage leading to plant-eating graviportal quadrupeds, *Spinophorosaurus* and other (but not all) sauropodomorphs show no reduction of the vestibular apparatus of the inner ear. This character-state is possibly a primitive retention in *Spinophorosaurus*, but due the scarcity of data it remains unclear whether it is also the case in the various later sauropods in which it is present or whether it has developed homoplastically in these taxa. Any interpretations remain tentative pending the more comprehensive quantitative analysis underway, but the size and morphology of the labyrinth of sauropodomorphs may be related to neck length and mobility, among other factors.

## Introduction

In 2006, a well-preserved sauropod skeleton was found in Niger as part of a cooperative project called PALDES (*PALeontología y DESarrollo*). The specimen was identified as a new species. Remes et al. [1] published a description and phylogenetic analysis of this taxon, which they named *Spinophorosaurus nigerensis*. *Spinophorosaurus* comes from the ‘Argiles de l'Irhazer’. This rock unit underlies the Tiourarén Formation, which is probably of latest Middle Jurassic age [2], and is most likely only slightly older than it (?Bathonian).

The braincase of *Spinophorosaurus* was collected along with the postcranial skeleton. It is provisionally housed at the Museo Paleontológico de Elche (Elche, Spain) under the specimen number GCP-CV-4229 and will eventually return to Niger, where it will be kept at the Musée National in Niamey. The aim of the present article is to offer a detailed osteological description of this braincase as well as digital reconstructions of the endocast and endosseous labyrinth of the inner ear based on CT scanning.

### Institutional abbreviations

AMNH, American Museum of Natural History, New York, USA; ANS, Academy of Natural Sciences, Philadelphia, USA; BP, Bernard Price Institute for Palaeontological Research, University of the Witwatersrand, Johannesburg, South Africa; CM, Carnegie Museum of Natural History, Pittsburgh, USA; GCP, Grupo Cultural Paleontológico de Elche, Museo Paleontológico de Elche, Elche, Spain; HMS, Houston Museum of Science, Houston, USA; ISI, Indian Statistical Institute, Kolkata, India; MB.R., Collection of fossil Reptilia, Museum für Naturkunde, Berlin, Germany; MCZ, Museum of Comparative Zoology, Harvard University, Cambridge, USA; MNN, Musée National du Niger, Niamey, Niger.

## Materials and Methods

In the comparisons, special emphasis will be placed on other African Jurassic sauropods for which the braincase is more or less adequately known: *Tazoudasaurus naimi* Allain et al., 2004 [3] (?Toarcian, Morocco), *Atlasaurus imelakei* Monbaron et al., 1999 [4] (?Bathonian, Morocco), *Chebsaurus algeriensis* Mahammed et al., 2005 [5] (?Callovian, Algeria), *Jobaria tiguidensis* Sereno et al., 1999 [6] (?Callovian, Niger), *Dicraeosaurus hansemanni* Janensch, 1914 [7] (Kimmeridgian, Tanzania), *Giraffatitan brancai* (Janensch, 1914) [7] (Kimmeridgian-Tithonian, Tanzania), and *Tornieria africana* (Fraas, 1908) [8] (Kimmeridgian-Tithonian, Tanzania). We concur with Remes [9] that the sauropod cranial specimens from Tendaguru most probably represent more than the three taxa suggested by Janensch [10], though we provisionally maintain this taxonomy pending a comprehensive systematic revision of this material. Unfortunately, detailed data on the braincases of *Atlasaurus* and *Jobaria* could not be obtained because the specimens are under study.

We based these comparisons on the examination of original fossil specimens (especially MB.R.2379.1-3 (formerly dd307), MB.R.2378.1-5 (formerly dd495), MB.R.2384.1-3 (formerly Y1), MB.R.2180.22.1-4 (formerly S66), MB.R.2386 (formerly k1), MB.R.2388.1-2 (formerly dd130) and MB.R.2387.1-4 (formerly dd316)), physical casts of the endocranial and labyrinth cavities (particularly MB.R.1916.1 (formerly dd307), MB.R.1917 (formerly dd495), MB.R.1918.2 (formerly Y1), MB.R.1919 (formerly S66), MB.R.1912 (formerly k1), MB.R.1915 (formerly dd130), MB.R.1913 (formerly dd316) and MB.R.2180.22.5 (formerly S66)), and the literature (mostly [4], [6], [10]–[13]).

We also drew on comparisons, particularly for the cranial endocast, with North American Jurassic (Kimmeridgian-Tithonian) sauropods, such as *Diplodocus longus* Marsh, 1878 [14] (especially AMNH 694, CM 11161, and CM 3452), *Camarasaurus lentus* (Marsh, 1889) [15] (CM 11338), and *Suuwassea emilieae* Harris et Dodson, 2004 [16] (ANS 21122), as well as the Nigerian Cretaceous (?Aptian) sauropod *Nigersaurus taqueti* Sereno et al., 1999 [6] (MNN GAD512) and the southern African Jurassic (Hettangian) basal sauropodomorph *Massospondylus carinatus* Owen, 1854 [17] (BP/1/4779).

To produce a three-dimensional reconstruction of the endocast of the cranial cavity and endosseous labyrinth of the inner ear, the specimen was scanned on a Yxlon CT Compact (Yxlon International, Hamburg, Germany) with a voltage of 210 kV and a current of 2.8 mA. The slice thickness was 0.5 mm, with an inter-slice spacing of 0.25 mm. The in-plane pixel size was 0.293 mm. The raw scan data were reconstructed using a bone algorithm. Data were output from the scanner in DICOM format and then imported into Amira v. 4.2 (Mercury-TGS, Chelmsford, MA, USA) for viewing, analysis, and visualization. The resulting 3D models were then imported into the 3D modelling software Maya 8.5 (Autodesk, San Rafael, CA, USA) for artefact removal, final rendering, and generation of the illustrations. The 3D PDF in the Supporting Information was generated by exporting the 3D models from Maya into Deep Exploration 5.5 (Right Hemisphere, San Ramon, CA, USA) and then Adobe Acrobat 9 Pro Extended (Adobe Systems Inc., San Jose, CA, USA). The data are archived at the Departamento de Paleobiología of the Museo Nacional de Ciencias Naturales-CSIC (Madrid, Spain) and at WitmerLab at Ohio University (Athens, OH, USA). Scan protocols were reported in Witmer et al. [18] for the other specimens mentioned here from which virtual endocasts were generated.

## Results

### Osteology

**Figure 1:**
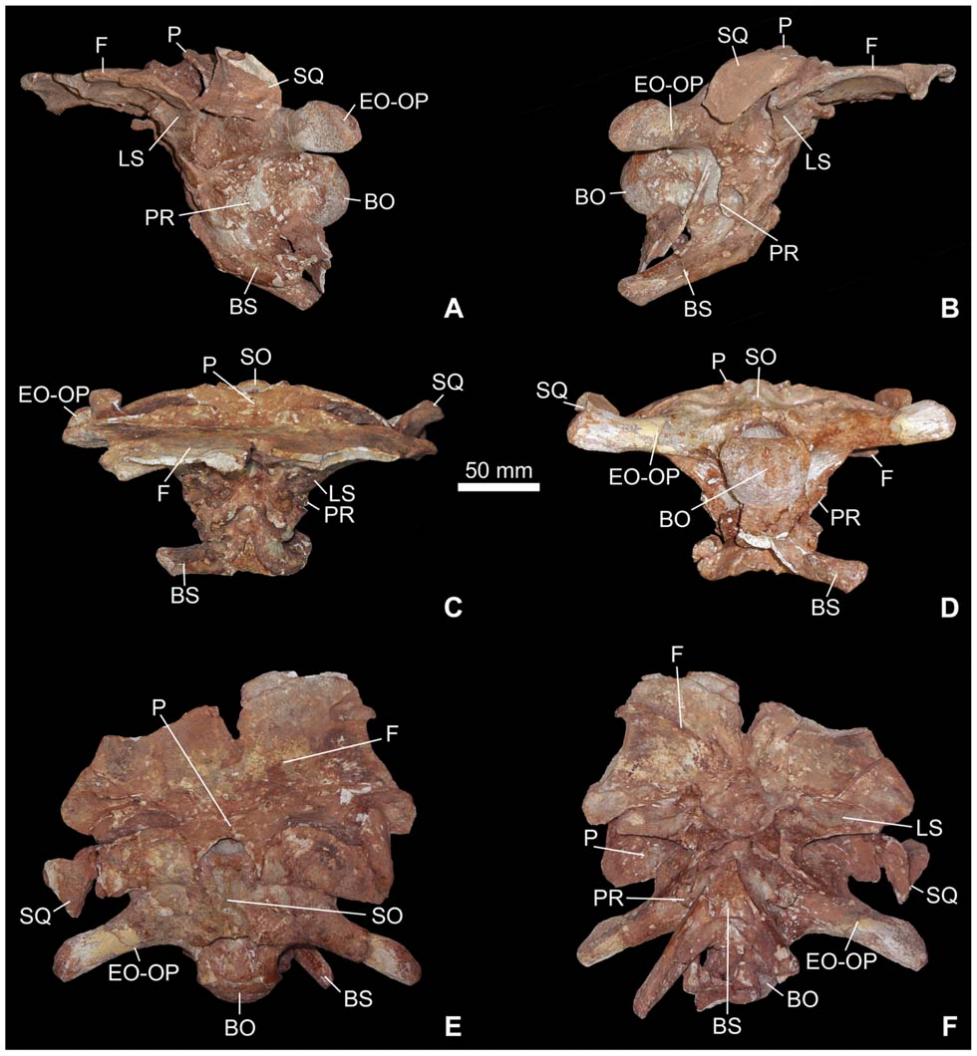
Photographs of the braincase of the sauropod dinosaur *Spinophorosaurus nigerensis* (GCP-CV-4229) from the Jurassic of Aderbissinat, Niger; in left lateral (A), right lateral (B), rostral (C), caudal (D), dorsal (A), and ventral (B) views. Abbreviations herein, in Figure 2, and Figure 3A–D: BO, basioccipital; BP, basipterygoid process; BS, basisphenoid; BT, basal tuber; C, columella; CA, crista antotica; CAR, carotid artery; CO, crista otosphenoidalis; CP, capitate process; CPC: craniopharyngeal canal vestigial pit; CT, crista tuberalis; CVCM, dorsal-head/caudal-middle-cerebral vein system groove; EO-OP, exoccipital-opisthotic; F, frontal; FM, fenestra metotica emplacement; FO, fenestra ovalis emplacement; FOM: foramen magnum; III, oculomotor foramen; LS, laterosphenoid; NC: nasal cavity recess; OBF: olfactory bulb fossa; OC, occipital condyle; OCV, orbitocerebral vein foramen; OR: orbital recess; P, parietal; PAF: proatlas facet; PFO, pituitary fossa emplacement; PIN, pineal foramen; PP, paroccipital process; PPF, postparietal fenestra; PR, prootic; PTF: posttemporal fenestra; RVCM, rostral middle cerebral vein foramen; SO, supraoccipital; SQ, squamosal; UTF, upper temporal fenestra; V, trigeminal foramen; VII, facial foramen emplacement; XII, hypoglossal foramen emplacement.

**Figure 2:**
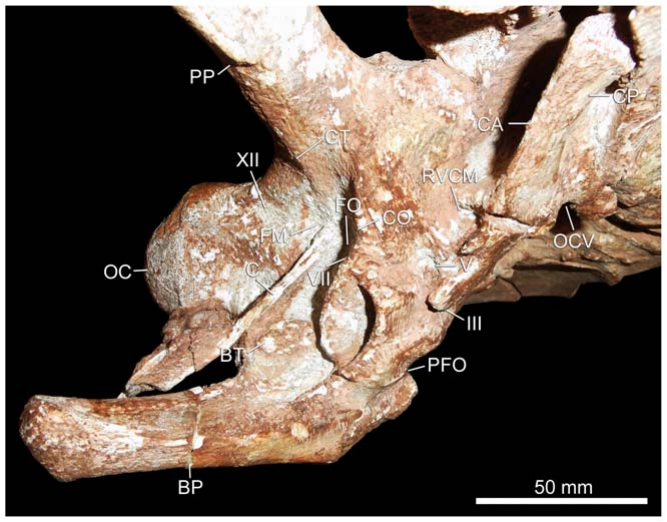
Close up photograph of the right sidewall of the braincase of the sauropod dinosaur *Spinophorosaurus nigerensis* (GCP-CV-4229) from the Jurassic of Aderbissinat, Niger.

**Figure 3:**
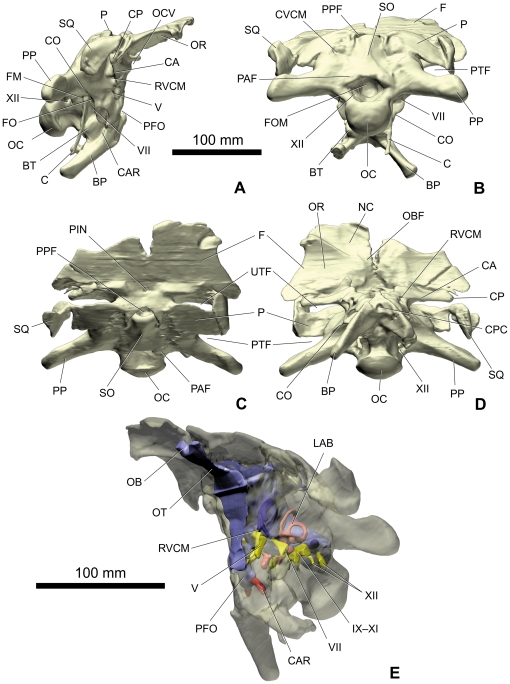
Volume-rendered CT images of the braincase of the sauropod dinosaur *Spinophorosaurus nigerensis* (GCP-CV-4229) from the Jurassic of Aderbissinat, Niger; opaque and unfilled in right lateral (A), caudal (B), dorsal (C), and ventral (D) views and semi-transparent with the endocast and associated structures in oblique view (E). Abbreviations in subfigure E and Figure 4: CAR, carotid artery; CBL, cerebellum; CE, cerebrum; CVCM, dorsal-head/caudal-middle-cerebral vein system; DE, dural expansion; IX, glossopharyngeal nerve; IX–XI, glossopharyngeal and vagoaccessory nerves; jug, jugular vein; LAB, labyrinth; MO, medulla oblongata; OB: olfactory bulb; OT, olfactory tract; pd, perilymphatic duct; PFO, pituitary fossa; RVCM, rostral middle cerebral vein; SIN, blind dural venous sinus of hindbrain; V, trigeminal nerve; VI, abducens nerve; VII, facial nerve; X–XI, vagoaccessory nerve; XII, hypoglossal nerve.

The braincase of *Spinophorosaurus* (Figures 1, 2, 3, S1, S2, S3) is incomplete but otherwise generally well preserved. It is rostrocaudally short and moderately deep in proportion but broad and of overall relatively large size. Most of the lateral and ventral wall of the braincase in front of the trigeminal foramen cannot be adequately interpreted, because the orbitosphenoids are displaced, having been crushed into the endocranial cavity.

### Frontal

No interfrontal suture is visible. A deep notch follows the midline of the conjoined frontals along their rostral half. If this character is genuine, it was possibly related to each nasal originally sending a prong of its caudomedial margin in between the frontals. This character is not common among sauropods, but it is suspected in *Nigersaurus* as well ([19]:fig. 1B). The dorsal surface of the frontals is fairly flat except for the lateral margins, which bear very discrete transverse wrinkles, as in *Camarasaurus* sp. (AMNH 545; Kimmeridgian, USA). The better preserved right frontal bears a deep notch in its rostrolateral area, presumably for reception of the prefrontal. The ventral surface of the frontals shows two large, shallow concavities. The most rostral one occupies the rostromedial corner of each frontal and represents the caudal (olfactory) region of the nasal cavity. The more caudal concavity, which is larger, occupies the caudolateral end of each frontal and represents the roof of the orbit. The straight crest that separates the two concavities attenuates medially as the frontal become thicker. The better preserved right frontal is rostrocaudally short and approximates a right-angled trapezoid in outline. A small, median, ovoid perforation (about 5.1×3.9 mm) between the frontals was interpreted by Remes et al. [1] as the pineal foramen (also known as the parietal, frontoparietal, postfrontal, and interfrontal foramen). Its irregular border makes it unclear, however, if it was really open in life. Witmer et al. [18] discussed the variability of this aperture in sauropods and its potential relationship with the underlying dural venous sinuses. For example, although a foramen in this location was identified in *Camarasaurus* by Chatterjee and Zheng ([20]:fig. 9.1B), Witmer et al. [18] noted that it was definitively absent in other specimens of the same taxon. The right frontal is about 103.1 mm wide at most (caudal margin) and 71.8 mm long rostrocaudally at the midline.

In contrast with the condition in *Spinophorosaurus*, the frontal does not contribute to the upper temporal fenestra in *Atlasaurus* ([3]:supplementary information) nor in *Dicraeosaurus*. The frontal of *Dicraeosaurus* contributes caudomedially to a large parietal foramen. In *Jobaria* ([6]:fig. 2A–B; [21]:appendices 1–2), the frontal appears to have been as short as in *Spinophorosaurus*.

### Parietal

The frontoparietal sutures are completely fused externally, but some vestiges are visible internally in the CT scan data. The frontals are defined caudally on the basis of the relatively sharp angle at their transition with the parietal. The midline interparietal suture is easily visible; it is not straight but interdigitating. The parietal table tilts rostroventrally, whereas the frontal extends rostrodorsally (the horizontal reference plane for the braincase is taken as the orientation when the lateral semicircular canals are held slightly inclined above the horizontal [18]). The parietals are very short rostrocaudally, as is typical for sauropods. Their rostrolateral prolongations delimit the medial half of the upper temporal fenestrae rostrally. The occipital wings (lateral extension of the parietal) take the form of an arched rectangle and completely border the upper temporal fenestrae caudally. Their caudolateral borders do not contact any bone where they form the dorsal margin of the posttemporal fenestrae. The upper temporal fenestrae are extremely short rostrocaudally, about four times shorter (rostrocaudally) than they are broad (mediolaterally). In dorsal view, they adopt a transversely lengthened ovoid outline. A short upper temporal fenestra was thought to be an attribute of sauropodomorphs more derived than *Shunosaurus* (?Bajocian, China) [22], but it is possibly also the condition in *Tazoudasaurus* [11]. The caudal margin of the parietal is marked by a large, median hemispheric notch. Because the supraoccipital is convex (arched caudally) in this zone, this produces a large postparietal opening between the two bones. Laterally, the contact of the parietal with the supraoccipital is not flush, the former does not fit into the latter but rather overlies it. As a result, the contact is straight (not interdigitating) and conducted vasculature between the endocranial cavity and the occipital region.

The frontoparietal suture is oblique in *Tazoudasaurus* ([11]:fig. 5E). In *Atlasaurus* [4], the dorsal margins of the upper temporal fenestrae do not slope laterally as in *Spinophorosaurus* and other sauropods. However, both in *Atlasaurus* ([3]:supplementary information) and *Spinophorosaurus*, the long axis of the upper temporal fenestrae is transverse and clearly greater than the maximum diameter of the foramen magnum. No postparietal opening occurs in *Atlasaurus* ([3]:supplementary information). *Jobaria* ([6]:fig. 2A–B; [21]: appendices 1–2) also lacks a postparietal aperture; the parietal instead appears to be pointed at this place. In *Dicraeosaurus* (MB.R.2379), the parietal is distinctly longer rostrocaudally than that of *Spinophorosaurus*, separated from the frontal by a distinct bulge-like step, and is characterized by the presence of two (not one) large openings in the cranial roof (parietal and postparietal fenestrae). An important difference between the braincase of *Spinophorosaurus* and that of *Giraffatitan* (MB.R.2180.22) is the much wider opening of the upper temporal fenestra in the latter; the caudal edge of the frontal and the dorsal border of the occipital wing of the parietal form an open angle in *Giraffatitan*, whereas these two rims are parallel in *Spinophorosaurus*. In *Tornieria* (MB.R.2386, MB.R.2387), the upper temporal fenestrae are less widely open than in *Giraffatitan*. They, however, differ from those in *Spinophorosaurus* in being caudolaterally oriented, whereas they are more perpendicular to the sagittal axis and more linear in this taxon. In *Omeisaurus* (?Bajocian, China) ([23]:fig. 8, pl. 1 fig. 1a, pl. 2 fig. 1), the elliptical upper temporal fenestra is not as short as in *Spinophorosaurus*.

### Supraoccipital

The supraoccipital is inclined rostrally, which confers to *Spinophorosaurus* a very low cranial roof. The limit of the supraoccipital with the exoccipital-opisthotic complex is difficult to discern, but it seems to have been arcuate, with the supraoccipital being narrowest at about its mid-height. The supraoccipital is marked by a triangular, median nuchal (occipital) crest. This bone constitutes apparently the central third of the dorsal margin of the foramen magnum. No epiotic is visible (this bone is probably completely fused with the supraoccipital, as is normally the case in braincases of adult archosaurs). The supraoccipital of *Spinophorosaurus* is about 60.4 mm wide at its base, 52.2 mm where it contacts the ventral border of the parietal, 51.3 mm in its narrowest part, and its midline is about 62.1 mm high (which is about twice the height of the foramen magnum).

**Figure 4:**
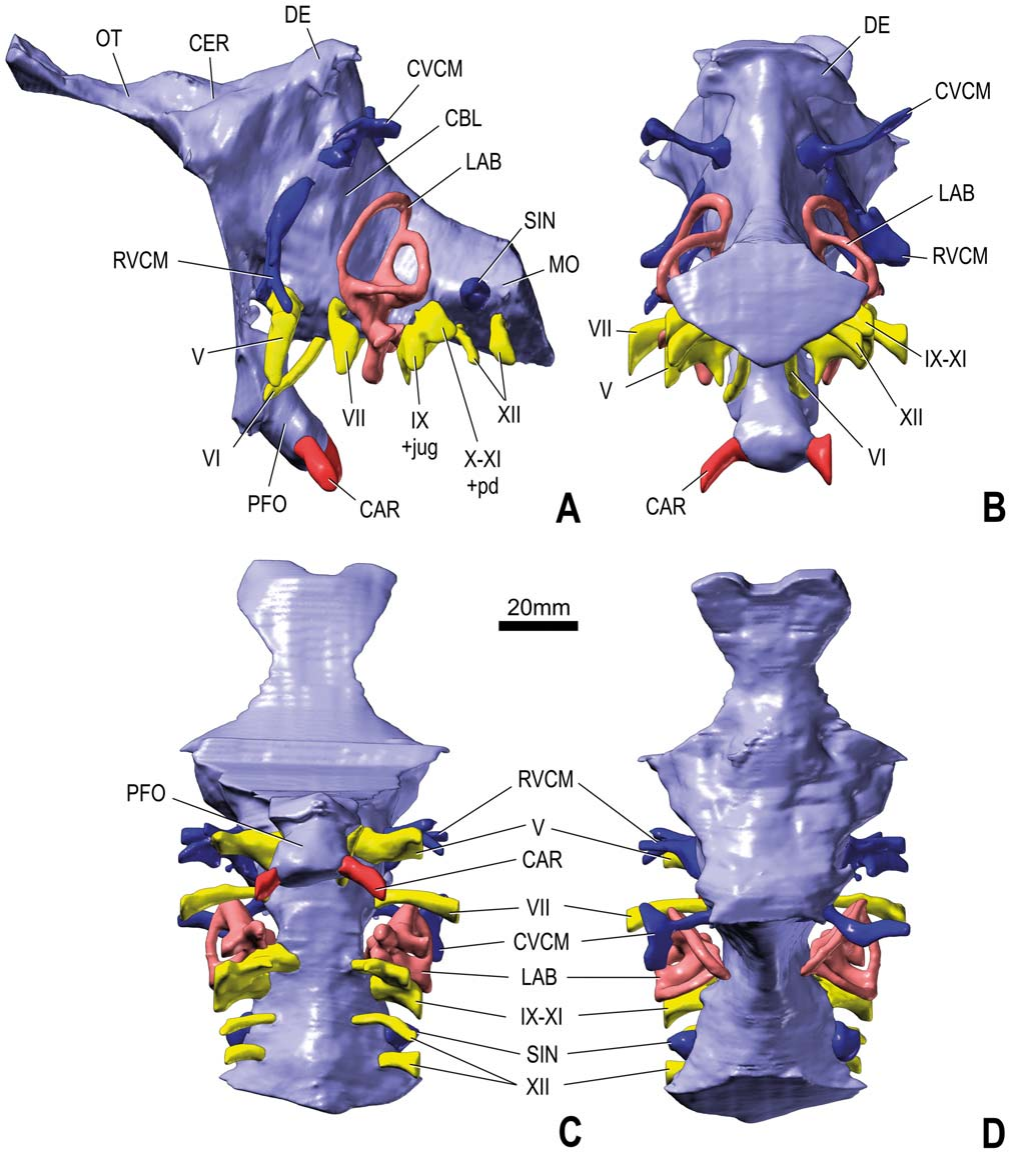
Cranial endocast, endosseous labyrinth, and some endocranial vascular structures of the sauropod dinosaur *Spinophorosaurus nigerensis* (GCP-CV-4229) from the Jurassic of Aderbissinat, Niger, derived from surface renderings of CT scan; in left lateral (A), caudal (B), ventral (C), and dorsal (D) views.

In *Tazoudasaurus* [11], the participation of the supraoccipital to the foramen magnum is more extensive than in *Spinophorosaurus*. A rostral inclination of the supraoccipital may be present in *Atlasaurus* ([4]:fig. b), similarly to the condition in *Spinophorosaurus*. However, the supraoccipital in *Atlasaurus* is not as high as it is in *Spinophorosaurus*. In *Jobaria* ([21]:appendices 1–2), the supraoccipital is about twice as high as the foramen magnum, just as in *Spinophorosaurus*. In *Dicraeosaurus* (MB.R.2378, MB.R.2379), the supraoccipital is more vertical than in *Spinophorosaurus*, and there is no overlap of the parietal onto the supraoccipital (but onto the exoccipital instead). In this taxon (MB.R.2379; appears lacking in MB.R.2378), two foramina related with the dorsal-head/caudal-middle-cerebral vein system deeply pierce the occipital plate on each side of the nuchal crest. A postparietal aperture also occurs in sauropods, such as *Dicraeosaurus* (MB.R.2379, MB.R.2378) and *Suuwassea* (ANS 21122), as well as in more basal sauropodomorphs, such as *Massospondylus* (BP/1/4779) and *Plateosaurus* (MB.R.1937; Norian, Germany). In *Giraffatitan* (MB.R.2180.22), there is no opening at the top of the supraoccipital. On the supraoccipital of *Giraffatitan*, a strong nuchal crest appears a little dorsal to the foramen magnum and acquires maximal prominence in the contact zone with the parietal. This crest, which is more marked in MB.R.2180.22 ([10]:fig. 7) than in MB.R.2384 ([10]:fig. 4), is much weaker in *Spinophorosaurus*. The contact zone between the top of the supraoccipital and the parietal in *Tornieria* (MB.R.2387) recalls that of *Spinophorosaurus* in that there is an opening and that the parietal slightly exceeds caudally the top of the supraoccipital. The nuchal crest is much weaker in *Tornieria* than in *Giraffatitan* but still a little stronger than in *Spinophorosaurus*. As in *Spinophorosaurus*, the supraoccipital is wider than high in *Omeisaurus*. However, its outline appears different ([23]:fig. 6), especially near the middle transverse plane, where it extends the farthest laterally, whereas it is narrow in *Spinophorosaurus*.

### Exoccipital-opisthotic

The exoccipital and opisthotic are co-ossified in a single complex (otoccipital), as is typical in most archosaurs. The limits of the exoccipital-opisthotic with the surrounding bones are not perfectly clear. It seems to contact the parietal dorsally, along the medial portion of the occipital wings of the latter. It forms most of the margin of the foramen magnum, from the lateral third of the dorsal border to about the lateral third of the ventral border. The foramen magnum is ovoid and wider than high (45.6×29.8 mm). The exoccipital-opisthotic complex indeed contributes to the occipital condyle to an extent close to what can be seen in *Camarasaurus* [24]. The caudal surface of the exoccipital-opisthotic complex is marked by well-defined articular facets medially, presumably for the proatlas. The paroccipital processes are oriented slightly caudoventrally. They are different from most other sauropodomorphs, including basal forms such as *Massospondylus* (BP/1/4779) and *Plateosaurus* (MB.R.1937), in being rostrocaudally thickened and stick-like rather than flattened, expanding only slightly dorsoventrally at their extremities. From the foramen magnum to their lateral tip, they are at least 93 mm long. The crista tuberalis is visible at the base of the paroccipital process but remains extremely low and disappears rapidly on the sidewall of the braincase, at the level of the crista interfenestralis. The CT data confirm that, as in other sauropods, the fenestra metotica (metotic fissure), which formed the exit of the glossopharyngeal and vagoaccessory nerves (IX, X–XI), open just caudal to the fenestra ovalis ( = fenestra vestibuli of Witmer et al. [18]). CT data also corroborate that a foramen visible on the left side of the braincase near the base of the paroccipital process is an opening for the caudal branch of the hypoglossal nerve (XII). They also reveal the position of a smaller, more rostroventral branch of the hypoglossal nerve.

In contrast with the condition in *Spinophorosaurus*, the suture between the exoccipital-opisthotic complex and basioccipital is interdigitating in *Tazoudasaurus* [11]. In caudal view, the paroccipital processes of *Atlasaurus* ([4]:fig. b; [25]:pl. VII fig. a) are horizontally oriented as in *Spinophorosaurus*. However, in the former they are also perpendicular to the long axis of the skull, whereas in the latter they project more caudally. In *Jobaria* ([6]:fig. 2A–B; [21]:appendices 1–2), the paroccipital processes are oriented transversely. In *Dicraeosaurus* (MB.R.2379), the paroccipital processes are complete and have an entirely different shape from that seen in *Spinophorosaurus*. They are flat and much more dorsoventrally expanded in a wing-like manner in the former, as in other sauropods, whereas they have a more rounded cross-section in the latter. In *Giraffatitan* (MB.R.2180.22), the paroccipital processes widen dorsoventrally (in a fan-like fashion) at their lateral tip, whereas in *Spinophorosaurus* this widening is much weaker. The paroccipital processes are also broadened at their distal extremities in *Tornieria* (MB.R.2388) but less so than in *Giraffatitan*. In this respect, the paroccipital processes of *Spinophorosaurus* resemble those of *Tornieria* more so than those of *Giraffatitan* but are longer and not as flat. Moreover, the foramen magnum is higher than wide in *Tornieria* (29.3×33.3 mm in MB.R.2387). In *Spinophorosaurus*, the paroccipital processes form a wider angle with the occipital condyle than in *Omeisaurus* ([23]:fig. 6, pl. 1 fig. 2, pl. 2 fig. 2). The paroccipital process of *Bellusaurus* (?Callovian, China) ([26]:fig. 2, pl. 4 fig. 1) seems to have been shorter than in *Spinophorosaurus*. In *Shunosaurus* [27], there are no posttemporal fenestrae.

### Basioccipital

There is no dorsal constriction of the neck of the occipital condyle, such that the articular surface of the occipital condyle is in gentle continuity with the dorsal surface of the basioccipital. However, laterally and ventrally, the neck is deeply concave in *Spinophorosaurus*. In caudal view, the occipital condyle is concave in outline dorsally. The basal tubera are moderate in size, forming blunt and rounded prominences that are closely in contact with the zone of the basipterygoid processes. The occipital condyle is 55.5 mm wide and about 41.5 mm tall.

In *Dicraeosaurus*, the basal tubera are narrow. In this taxon and in contrast with *Spinophorosaurus*, the articular surface of the occipital condyle curves rostrally in its ventral part, weakly in MB.R.2379 but farther in MB.R.2378. In *Tornieria*, the occipital condyle is particularly small (in MB.R.2387: 35.3 mm wide×27.9 mm tall). In *Shunosaurus* ([27]:fig. 7), the basal tubera are proportionally much more developed than in *Spinophorosaurus*.

### Basisphenoid-parasphenoid

Due to the incompleteness of the specimen, the dorsal portion of the dorsum sellae is visible in a rostral view of the braincase. This effectively marks about the rostral limit of the preserved basisphenoid. The pituitary ( = hypophyseal) fossa, which is roughly ovoid in section (13.0 mm wide×9.6 mm high), is directed caudoventrally, as is usually the case among sauropods [18], [28]. The basipterygoid processes are elongate but only moderately so. They are straight, subtriangular in cross-section, and extend strongly caudally and moderately ventrally (i.e., they form a very acute angle with the skull roof). In ventral view, the processes diverge from the long axis of the skull by a little more than 30°. A median pit is located ventrally between their bases, and CT data show that it is blind. This pit is likely the vestige of the craniopharyngeal canal [12], [29]–[30], formed during embryogenesis of the adenohypophysis, and is a common feature of archosaurs. The crista otosphenoidalis ( = crista prootica) connects the rostral surface of the paroccipital process with the distal tip of the basipterygoid process, passing in turn across the exoccipital-opisthotic, prootic, and basisphenoid, and separating the middle ear and adductor chamber domains (see [31]). The crest continues caudoventrally on the basisphenoid, where it then blunts and parallels the lateral side of the basipterygoid process, at the end of which it becomes sharp again. The parasphenoidal rostrum is broken at its base where it is triangular in cross section as in most sauropods. It is impossible to delimitate the parasphenoid. It is probable that the basisphenoid is actually a basisphenoid-parasphenoid complex, as is generally seen in sauropods and most other diapsids.

The orientation and extension of the basipterygoid processes are similar in *Spinophorosaurus* and *Atlasaurus* ([4]:fig. b–c). In both, they diverge from one another in a V-shaped fashion, rather than in a somewhat more U-shaped one as in *Chebsaurus* [13]. The processes are, however, more robust in *Spinophorosaurus* than in *Atlasaurus*. The basipterygoid processes of *Chebsaurus* are distinctly different from those of *Spinophorosaurus* in that they do not extend caudally in any manner but project essentially ventrolaterally ([13]:fig. 4A–C, F–G). Thus, they would not have concealed the basal tubera in ventral view as it is the case in *Spinophorosaurus*. The minute foramen that pierces the base of each basipterygoid process in *Chebsaurus* ([13]:fig. 4A) appears to be absent in *Spinophorosaurus*. In *Dicraeosaurus* (MB.R.2379), the basipterygoid processes are strongly elongate, thin, and extend essentially ventrally and a bit rostrally, whereas they are significantly shorter in *Spinophorosaurus* and prolong mostly caudally and only a little ventrally. This is the most obvious difference between the braincase anatomy of *Dicraeosaurus* and that of *Spinophorosaurus*. In *Giraffatitan* (MB.R.2180), the basipterygoid processes also are oriented nearly entirely ventrally and much less laterally than in *Spinophorosaurus*. In the latter, the caudal extension of the basipterygoid processes is also much more marked than in the former. The bases of the basipterygoid processes are rooted much more dorsally in *Tornieria* (MB.R.2386, MB.R.2388, MB.R.2387) relative to *Spinophorosaurus*, which is related to the comparatively shallow basisphenoid in *Spinophorosaurus*. In *Shunosaurus* ([27]:fig. 7), the stout basipterygoid processes are shorter than in *Spinophorosaurus*. Moreover, they are subcircular in section at their bases, not oriented caudally, and diverge from one another in a widely open U-shaped fashion. In *Chebsaurus*, the cross section of the parasphenoid is elliptical rather than triangular at its base ([13]:fig. 4A).

### Prootic

The prootic is a rostrocaudally short but dorsoventrally deep bone that is situated rostral to the basioccipital. The prootic is marked by a sharp crest, the crista otosphenoidalis, which emerges near the base of the paroccipital process and continues ventrally. In the region of the facial foramen within the prootic, the crista otosphenoidalis is bifurcated, which is unusual if not unique. Full preparation of the sidewalls of the braincase was not possible, so the position and identification of the foramina could not be precisely determined without CT scan data. As in other sauropod dinosaurs [18], there is a single trigeminal foramen. This aperture is situated on the junction of the prootic with the laterosphenoid and in close proximity to other openings related to the rostral middle cerebral vein complex. It is not as largely open as could be expected and its external outline differs from the heart-shaped trigeminal foramina of some sauropods such as *Dicraeosaurus* (MB.R.2379) and cf. *Cetiosaurus* (Bathonian, United Kingdom) [22]. The facial foramen appears to be situated caudal to the crista otosphenoidalis, just dorsal to its bifurcation. Whether or not an accessory ramus of the facial nerve emerged from the braincase deep in the cavity formed by the bifurcation of the crista otosphenoidalis is uncertain. The fenestra ovalis is situated close to the facial foramen, the proximal extremity of the preserved (right) columella (see below) being a little displaced from it.

The prootic-basisphenoid complex of *Dicraeosaurus* (MB.R.2379) is peculiar in that the preotic pendants (the attachment sites for the protractor musculature [32]) are relatively large ovoid lamellar processes (the dorsolateral processes of Salgado and Calvo [33]) at about the same level as the basal tubera but more rostrally. In *Spinophorosaurus*, on the other hand, the preotic pendants are almost absent, forming just roughened areas on the rostral surface of the otosphenoidal crest. In *Giraffatitan* (MB.R.2180.22), the course of the crista otosphenoidalis is simpler than in *Spinophorosaurus*, in that it is in continuity with the rostroventral border of the paroccipital process. Thus, *Giraffatitan* lacks a deep bifurcation. In *Tornieria* (MB.R.2388), the crista otosphenoidalis is more marked dorsally (namely, in the zone of the base of the paroccipital processes) and the right (and presumably originally the left also) trigeminal foramen is single internally but split externally by a tiny strip of bone.

### Laterosphenoid

The laterosphenoid is a rostrocaudally short bone. As preserved, it rostrally borders the trigeminal foramen and caudally an orbitocerebral vein opening and the oculomotor foramen. The laterosphenoid is noteworthy in its relatively stout, fairly straight capitate process, which has an acute caudoventral border. This edge extends ventrally as the crista antotica, which essentially ends at the oculomotor foramen located between the laterosphenoid and the internally displaced orbitosphenoid. The capitate process is ovoid in distal cross section (8.4×20.8 mm). Its rounded extremity fits in the caudal border of the frontal, where this bone articulated with the postorbital.

The capitate process of the laterosphenoid is relatively (as well as absolutely), much smaller in *Dicraeosaurus* (MB.R.2379) than in *Spinophorosaurus*. In addition, in *Dicraeosaurus* (MB.R.2379) the capitate process points clearly caudally. In *Giraffatitan*, the capitate process (MB.R.2180.22, MB.R.2223, MB.R.2384) is somewhat more lamellar (‘sheet-like’) than that in *Spinophorosaurus*. The crista antotica extends ventrally from the capitate process of the laterosphenoid and passes between the external foramina for the oculomotor and trigeminal nerves. In *Tornieria* (MB.R.2388), the ventral extension of the capitate process appears less marked than in *Spinophorosaurus*.

### Squamosal

The squamosals are not preserved in their entirety. They are loosely attached to the braincase and both appear to have been displaced to some extent. As preserved, they contact the extremities of the lateral wing of the parietal but not the paroccipital processes. They are presumed to have articulated in life with the postorbital (which was not found articulated with the braincase) and, in so doing, to have closed the upper temporal fenestra laterally.

### Columella

An accessory element is embedded in the matrix on the right prootic caudal to the crista otosphenoidalis. It is a delicate, fairly straight bony rod, oval in cross section, which is incomplete distally but almost 69.3 mm long and 2.4 mm in diameter. It is undoubtedly a remnant of the columella. The proximal end is almost in natural articulation, pointing toward the fenestra ovalis, where it widens slightly into a footplate. This is especially remarkable as the columella is only known in a few sauropod taxa [24], [34], [35]. The preservation of this element in a largely disarticulated skull is surprising.

### Paleoneuroanatomy

The first virtual cranial cavity endocast of a dinosaur was generated from CT scans more than a decade ago [36]–[37]. This method has now supplanted traditional and potentially risky techniques, such as physical endocasts, although the latter still have utility. The endocast of the intracranial space of sauropodomorphs has been a focus of study for almost a century, and a number of important articles have been published (e.g., [28] and references therein and [12], [18]–[20], [22], [24], [27], [30], [38]–[40]). The significance of *Spinophorosaurus* is that most of the previous articles focus on relatively advanced neosauropods, and thus, as a basal eusauropod, *Spinophorosaurus* can shed new light on trends in sauropod evolution. For the sake of ease of description, we will refer to the reconstructed digital casts of bone-bounded spaces that housed soft-tissue structures as if they were the structures themselves (e.g., “trigeminal nerve” instead of “digital cast of trigeminal canal”).

**Figure 5:**
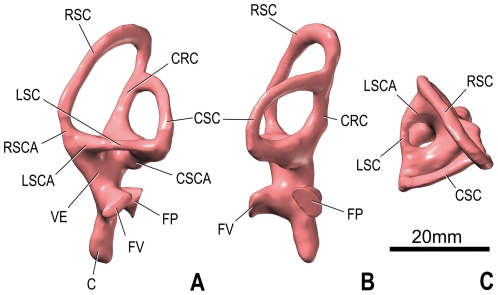
Endosseous labyrinth of the left inner ear of *Spinophorosaurus nigerensis* (GCP-CV-4229) reconstructed from CT scan; in lateral (A), caudal (B), and dorsal (D) views. Orientations were determined based on orientation of the labyrinth within the braincase and with the lateral semicircular canal placed horizontally. Abbreviations: C, cochlea ( = lagena); CRC, crus commune; CSC, caudal ( = posterior) semicircular canal; CSCA, ampulla of caudal semicircular canal; FP, fenestra perilymphatica ( = round window); FV, fenestra vestibuli ( = oval window); LSC, lateral ( = horizontal) semicircular canal; LSCA, ampulla of lateral semicircular canal; RSC, rostral ( = anterior) semicircular canal; RSCA, ampulla of rostral semicircular canal; VE, vestibule of inner ear.

Despite difficulties in discriminating the densities of the bone and matrix, the CT data resulted in a very faithful rendering of the cranial endocast and endosseous labyrinth (Figures 3, 4, 5, S1, S2, S3). Due to the imperfect preservation of the braincase (displacement of the orbitosphenoids into the cranial cavity, etc.), the rostroventral part of the endocast is missing. As a consequence, the position and configuration of the optic (II) and trochlear (IV) nerves could not be determined. The foramina for the oculomotor nerve (III) and the orbitocerebral vein are largely incomplete (Figure 2) and, therefore, these structures were not reconstructed.

### Brain

The endocast of *Spinophorosaurus* recalls that of other sauropods by a number of characters, such as the well-marked pontine and cerebral flexures (both about 45°) and the presence of a well-defined, large and oblong pituitary fossa (pendant caudally at about 35° from the horizontal). It is primitive in having the arrangement of the regions of the brain presumably obscured by the spaces that housed relatively thick meninges and extensive dural venous sinuses. This trait is the generalized condition in sauropods [18], although the meninges (and dural sinuses) appear to have been much thinner in another Nigerian sauropod, *Nigersaurus* [19], as well as possibly in some titanosaurs, as judged by the greater distinctness of the brain regions on the endocasts of these taxa. Caudodorsal to the cerebral region, a mushroom-shaped dural expansion sends two small wings laterally and communicates with the exterior of the braincase through the aperture between the parietal and the supraoccipital (postparietal fenestra). The dural expansion is a prominent venous feature of the endocasts of many sauropods (e.g., [18], [30]). This development is considerably less significant in *Spinophorosaurus* than in *Dicraeosaurus* ([10]:pl. 13 figs 6–7), but its morphology is very similar to that of *Massospondylus* and *Camarasaurus* ([18]:fig. 6.8; [19]:fig. 1G), although it does not breach the skull (i.e., there is no fenestra) in the latter. The small cerebrum is still relatively easily discernible and is connected with relatively large olfactory bulbs by short olfactory tracts. However, the margins or contours of the cerebellum and optic lobes cannot be discriminated, as is typical for sauropods [18]. The brainstem is somewhat lengthened, with a notable space between the trigeminal nerve and the otic region.

### Cranial Nerves

**Figure 6:**
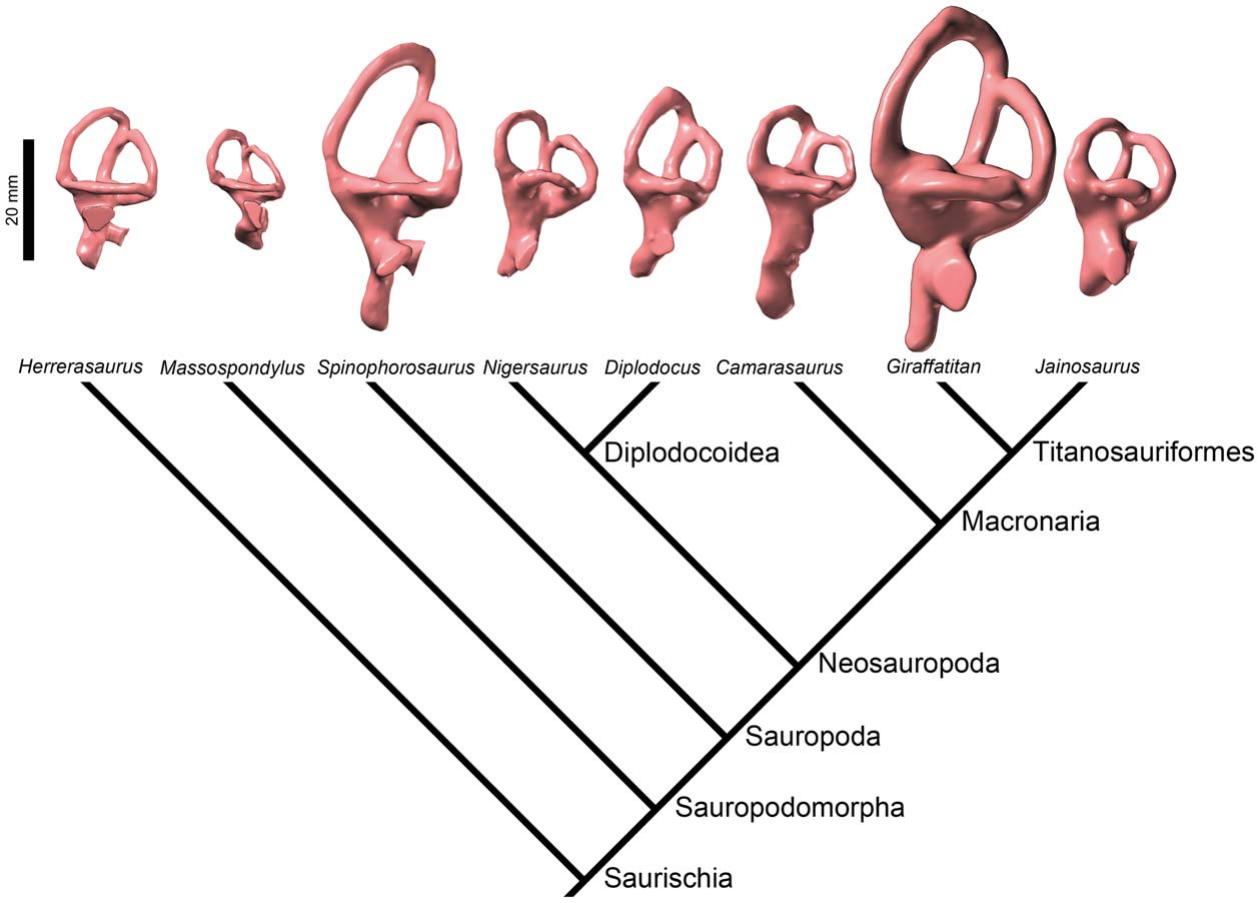
Endosseous labyrinths of the left inner ears of some saurischian taxa derived from surface renderings of CT images, displayed on a cladogram. From left: the basal theropod *Herrerasaurus ischigualastensis* (MCZ 7063), the basal sauropodomorph *Massospondylus carinatus* (BP/1/4779), the basal eusauropod *Spinophorosaurus nigerensis* (GCP-CV-4229), the basal diplodocoid *Nigersaurus taqueti* (MNN GAD512), the derived diplodocoid *Diplodocus longus* (CM 3452), the basal macronarian *Camarasaurus lentus* (CM 11338), the basal titanosauriform *Giraffatitan brancai* (MB.R.2180.22.1-4), and the titanosaurian *Jainosaurus septentrionalis* (ISI R162). With respect to that of other sauropods, the vestibular system of *Spinophorosaurus* is highly remarkable in its elongate semicircular canals and its overall large dimensions (both absolutely and in relation to body size).

As usual, the trigeminal nerve (V) is the largest of the cranial nerves. However, in *Spinophorosaurus* the differences of size between it and the other cranial nerves is much less marked than in other taxa, such as *Camarasaurus* ([18]:fig. 6.8) and *Giraffatitan* ([12]:figs 1–2). The nerve emerges ventrolaterally out of a bulge of the endocast that probably is related to the presence of a large trigeminal ganglion at this place in the dura mater. However, there is no evidence for the division of this nerve into a rostral (ophthalmic, V_1_) and a caudal (maxillomandibular, V_2–3_) ramus. This is somewhat surprising because a separation of these rami within the sidewall of the braincase is revealed by the outer outline of the prootic foramen in both cf. *Cetiosaurus* ([22]:fig. 3) and *Shunosaurus* ([27]:fig. 7A), taxa that are supposed to be more primitive and more derived, respectively, than *Spinophorosaurus* ([1]:fig. 6A). However, the basal sauropodomorphs *Plateosaurus* ([38]:fig. 7B–E) and *Massospondylus* ([19]:fig. 1G) exhibit a single trigeminal branch, suggesting that the condition in *Spinophorosaurus* may well be the primitive state.

The abducens nerve (VI) is relatively thick. As in other sauropods (e.g., cf. *Cetiosaurus* ([22]:fig. 6), *Apatosaurus* ([30]:fig. 7), *Giraffatitan* ([12]:fig. 1)), it emerges ventrally out of the endocast (near the pontine flexure) by traversing the dorsum sellae rostroventrally and thereby penetrates the pituitary space. The pituitary fossa is not fully preserved in *Spinophorosaurus*, but enough of it remains to see that the abducens nerves entered it relatively laterally at about its mid-length.

The facial nerve (VII) emerges from the endocast dorsal and slightly caudal to the abducens. As in reptiles in general, this nerve is small in diameter in sauropods (e.g., *Diplodocus* ([18]:fig. 6.9; [39]:fig. 2)), although that of *Spinophorosaurus* is not especially small. It passes first laterally and then slightly caudally.

The vestibular and acoustic branches of the vestibulocochlear nerve (VIII) penetrate the medial side of the braincase sidewall at mid-distance between the medial opening of the facial canal and the fenestra metotica. The inner ear itself is described below.

The glossopharyngeal nerve (IX) is generally thought (e.g., [18]) to have occupied the ventral half of the fenestra metotica together with the internal jugular vein, which represented a subsidiary route of drainage of blood from the endocranial cavity. It appears that the glossopharyngeal nerve entered the braincase sidewall through the jugular foramen, which was separated from the vagoaccessory by a bony strut on the medial side of the braincase (this bar appears to have been stronger on the left size than on the right). However, the two nerves left the braincase through a single elongate opening (the fenestra metotica) on both sides.

The vagoaccessory nerve (X–XI) exited the braincase through the dorsal half of the fenestra metotica. As for the glossopharyngeal nerve and other structures, the diameter of this nerve on the endocast probably does not reflect its original size since other tissues (e.g., perilymphatic duct) passed through the same foramen.

The hypoglossal nerve (XII) emerges from the endocast (medulla oblongata) as two separate rami, which pass ventrolaterally. The rostral ramus is smaller in diameter than the caudal ramus, as seen in specimens of other sauropod taxa (e.g., cf. *Cetiosaurus* ([22]:fig. 6), *Diplodocus* ([18]:fig. 6.9), *Camarasaurus* ([18]:fig. 6.8)).

### Endocranial Vasculature

The left and right internal carotid arteries penetrate into the braincase dorsal to the root of the basipterygoid processes and emerge in the brain cavity at the ventral tip of the pituitary fossa, as usual in dinosaurs [18], [28]. Because the carotid arteries constitute the main supply of blood for the brain, their diameter is large. In *Spinophorosaurus*, these divisions of the common carotid artery are well separated from one another when they reach the pituitary fossa. This configuration, which is common in sauropods, contrasts with that in some theropods (e.g., Tyrannosauridae ([42]:fig. 3B, E, K, N)). After having entered the pituitary fossa, each internal carotid artery divided into a rostral and a caudal portion. The rostral one sent a branch outward again in the orbital region, whereas the caudal one united with its counterpart as a single basilar artery that ran caudally beneath the brain. The basilar artery may have gone through the median canal that connects the pituitary space with the braincase cavity between the trigeminal and abducens nerves.

The dorsal-head/caudal-middle-cerebral vein system is evidenced by a strong dorsolateral projection that emerges in the middle part of the endocast near the dorsal border. This vein bifurcates on the left side of the endocast. The caudal middle cerebral vein opens onto the occiput at foramina in the zone of suture between the supraoccipital, parietal, and exoccipital-opisthotic bones, as is typically the case in many archosaurs [31]. Tributaries of the vein drain the osseous tissue. The caudal middle cerebral vein is continuous with the transverse sinus, which is visible on the endocast (more clearly on the right side) as a rounded ridge. From the ventrolateral end of the transverse sinus, the rostral middle cerebral vein passes through the laterosphenoid bone to emerge in apertures dorsal to the trigeminal foramen. The presence of a dorsal-head/caudal-middle-cerebral vein system appears primitive within sauropods, as it is present in basal sauropodomorphs, such as *Massospondylus*, as well as conservative neosauropods, such as *Camarasaurus* ([18]:fig. 6.8; [19]:fig. 1G), but is reduced in some derived diplodocoids and titanosauriforms ([19]:fig. 1G; [41]:fig. 7). In most neosauropods, the transverse sinus reaches the trigeminal foramen and therefore the trigeminal foramen also transmitted venous blood. In these cases, there is no separate rostral middle cerebral vein [18].

A blind dural venous sinus of the hindbrain is located just dorsal to the hypoglossal canals. A homologous structure, the posterior cerebral vein, is present in *Giraffatitan* ([12]:figs 2–3), but most sauropods lack any venous development at this place (e.g., [18]:figs 6.8–6.9). In contrast, it is common in theropods (e.g., [37]:fig. 2; [41]:fig. 1). The vein is supposed to have drained the longitudinal dural venous sinus, as in *Sphenodon* [42], and to have become blind after closure of a foramen in the exoccipital-opisthotic during early ontogeny.

### Inner Ear

The vestibular apparatus is very well developed. The crus commune is slightly curved caudally. The rostral semicircular canal is elevated significantly more dorsally than the caudal one (as in most archosaurs) and it is straight in its middle portion. In contrast, the caudal semicircular canal and the lateral semicircular canal, which are shorter, are arcuate all along their length. All three semicircular canals are relatively long and proportionally slender in comparison to most other sauropods. The oval window is indeed oval in outline on the left side but rather triangular on the right side. The lagena (cochlear duct) curves very slightly caudally at its tip.

## Discussion

Being less subjected to rapid evolutionary changes related to feeding or locomotion, braincases are generally regarded as being more ‘conservative’ and therefore useful for higher-level phylogenetic inferences. The braincase of *Spinophorosaurus* can generally be distinguished from that of the other African Jurassic sauropods. In particular, the osteological differences between the braincase of *Spinophorosaurus* and that of *Giraffatitan* are numerous and marked (e.g., more widely open upper temporal fenestrae in the latter; see above). The braincase of *Spinophorosaurus* is also clearly distinguished from that of *Dicraeosaurus* (e.g., presence of enlarged preotic pendants in the latter; see above). Likewise, the braincase of *Spinophorosaurus* is longer rostrocaudally than that of *Tornieria*; thus in the latter taxon (MB.R.2386, MB.R.2387), the caudal border of the interfrontal suture is directly dorsal to the base of the basipterygoid processes. From a dimensional viewpoint, the braincase of *Spinophorosaurus* is clearly larger than that of *Tornieria*, a little larger than that of *Dicraeosaurus* and smaller than that of *Giraffatitan*. In some characters, such as the configuration of the parietal, *Spinophorosaurus* appears more derived than *Tazoudasaurus* [11]. Despite numerous differences, the braincase of *Spinophorosaurus* appears to most closely resemble that of *Atlasaurus* ([4]:b–c) in its ‘lowness’ (‘shallowness’, dorsoventral compression) as well as in the configuration of the basipterygoid processes. Both the overall depth of the braincase and the arrangement (length, shape, and orientation) of the basipterygoid processes are variable among sauropods. We suggest that this may have phylogenetic significance. *Bellusaurus sui* positioned close to both *Jobaria* and *Atlasaurus* within neosauropods in the phylogenetic analysis of Upchurch et al. [43]. Regrettably, very limited information on the braincase of *Bellusaurus* is presently available [26]. In contrast, Royo-Torres et al. [44] found in a subsequent analysis that *Atlasaurus* is one node more derived than *Jobaria* in the lineage leading to neosauropods. Recently, Läng and Mahammed [13] suggested that *Atlasaurus* is close to *Haplocanthosaurus* (?Kimmeridgian, USA) within neosauropods. Unfortunately, the braincase of the latter taxon is not known.

As detailed above, the paleoneuroanatomy of *Spinophorosaurus* is, in some ways, intermediate between that of basal sauropodomorphs and that of neosauropods. It is typical of a generalized sauropod in a number of characters, such as a very long pituitary fossa that extends ventrally beyond the level of the ventral border of the brainstem, but it looks primitive in the relative slenderness of the semicircular canals. Indeed, theropods, the sister-group of sauropodomorphs, have elongated rather than bulky semicircular canals in general ([18]; [41]:figs 4, 8; Figure 6) and the basal sauropodomorph *Massospondylus* has also long canals (Figure 6). However, the “reasonable reconstructions” of the semicircular canals of the basal sauropodomorph *Plateosaurus* by Galton ([38]:fig. 7S) are short. Within sauropods, the diplodocoid *Diplodocus* has semicircular canals of medium thickness, whereas those of *Nigersaurus* are somewhat slenderer (Figure 6). *Camarasaurus* has especially short semicircular canals (Figure 6). The morphology of the vestibular apparatus of the titanosauriform *Giraffatitan* ([12]:fig. 2; Figure 6) is comparable to that of *Spinophorosaurus*, but many (but not all) more derived members of the clade have short semicircular canals ([45]:fig. 4; [46]:fig. 3; Figure 6). Quantification of these often subtle differences is underway and will be the subject of the dedicated study comprising even broader taxonomic sampling.

As suggested above, there is a significant amount of morphological homoplasy in the labyrinth of sauropodomorphs, complicating delineation of clearly directional evolutionary trends. Given that the semicircular canals sense angular acceleration of the head, their morphology and size are expected to be closely related to locomotor agility and neck mobility and this has been verified empirically in a variety of taxa (see e.g., [47]–[49]). Thus, the primate *Eulemur mongoz*, which is able to run quadrupedally along the tops of tree limbs and jump from one tree to another, has elongate semicircular canals (i.e., with large radii of curvature), whereas the sloth *Bradypus tridactylus*, which is excessively slow and unable to walk, has a contracted labyrinth ([50]:pls 8, 27). Similarly, in birds, the bony semicircular canals tend to be longer and more slender in deft fliers like the raven *Corvus corax*, whereas they are shorter and thicker in less aerobatic fliers such as the duck *Anas platyrhynchos* ([51]:figs 3–4, 16; see also [52]:tab. 7; [53]).

It is unlikely that any sauropod (nor any large, pillar-legged basal sauropodomorph) would qualify as a physically nimble animal. Nonetheless, sauropods generally had far more flexible necks and, therefore, greater natural range of movement of their relatively small heads than previously thought [54], [55]. This might account for the well-developed labyrinths of some sauropods, but actually the canals are relatively reduced in most species. The fact that virtually all sauropods were of comparable bauplan, with a small head mounted at the end of a long neck (see [56] for a possible exception), suggests that neck mobility (which admittedly may have varied by species) may not fully explain the highly plastic nature of labyrinth evolution in the group. Another factor might pertain to the neurological relationships of the vestibular apparatus to coordination of eye movements and the vestibulo-ocular reflex (see e.g., [57]). That is, it is possible that differences in semicircular canal sizes among sauropods may reflect differences in the importance of gaze stabilization mechanisms and/or visual tracking movements. It has been suggested that the typically small size of the vestibular apparatus in most sauropods, certainly in comparison to the larger canals of theropods, may have resulted, at least in part, from less reliance on highly coordinated and/or rapid visual tracking movements in sauropods [18]. By extension, apparent expansion of the vestibular apparatus in some sauropods may signal a great importance of vision and coordinated movements of the eyes, head, and neck in those species. In truth, interpretation of these differences will remain complicated until we have a better understanding of the quantitative scaling of vestibular attributes in sauropods. But moreover, controversy remains surrounding the fundamental biophysical mechanisms of the vestibular apparatus and the behavioral significance of interspecific differences in semicircular canal attributes in extant vertebrates (see e.g., [58]–[60]), and further experimental studies hopefully will likewise shed light on extinct taxa such as sauropods, as well.

## Supporting Information

Figure S1 **Interactive visualization made from the CT scan of the braincase of the sauropod dinosaur** ***Spinophorosaurus nigerensis*** **(GCP-CV-4229) from the Jurassic of Aderbissinat, Niger (small file).**
(PDF)

Figure S2 **Interactive visualization made from the CT scan of the braincase of the sauropod dinosaur** ***Spinophorosaurus nigerensis*** **(GCP-CV-4229) from the Jurassic of Aderbissinat, Niger (medium file).**
(PDF)

Figure S3 **Interactive visualization made from the CT scan of the braincase of the sauropod dinosaur** ***Spinophorosaurus nigerensis*** **(GCP-CV-4229) from the Jurassic of Aderbissinat, Niger (large file).**
(PDF)
