# Supplementary figures and images for: The Braincase of the Basal Sauropod Dinosaur Spinophorosaurus and 3D Reconstructions of the Cranial Endocast and Inner Ear
Source: PLoS One. 2012 Jan 17;7(1):e30060. doi: 10.1371/journal.pone.0030060 (PMC3260197; doi:10.1371/journal.pone.0030060)

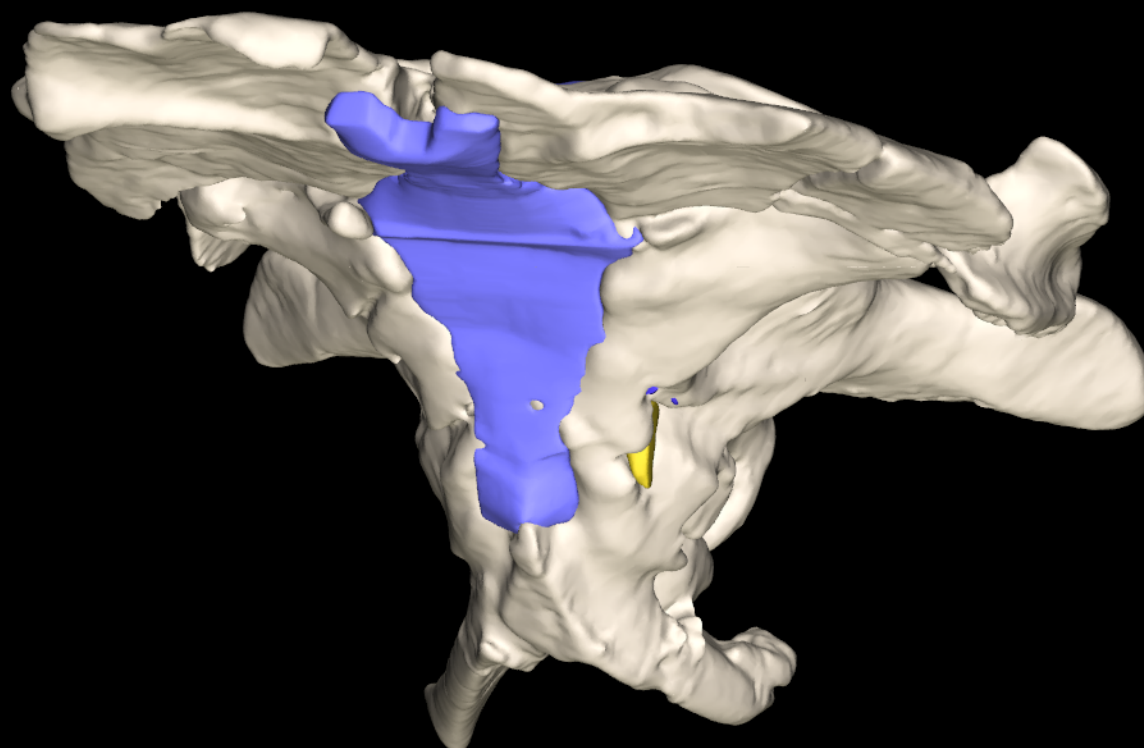

Supplement: Figure S1 — Interactive visualization made from the CT scan of the braincase of the sauropod dinosaur Spinophorosaurus nigerensis (GCP-CV-4229) from the Jurassic of Aderbissinat, Niger (small file). (PDF) [file pone.0030060.s001.pdf]

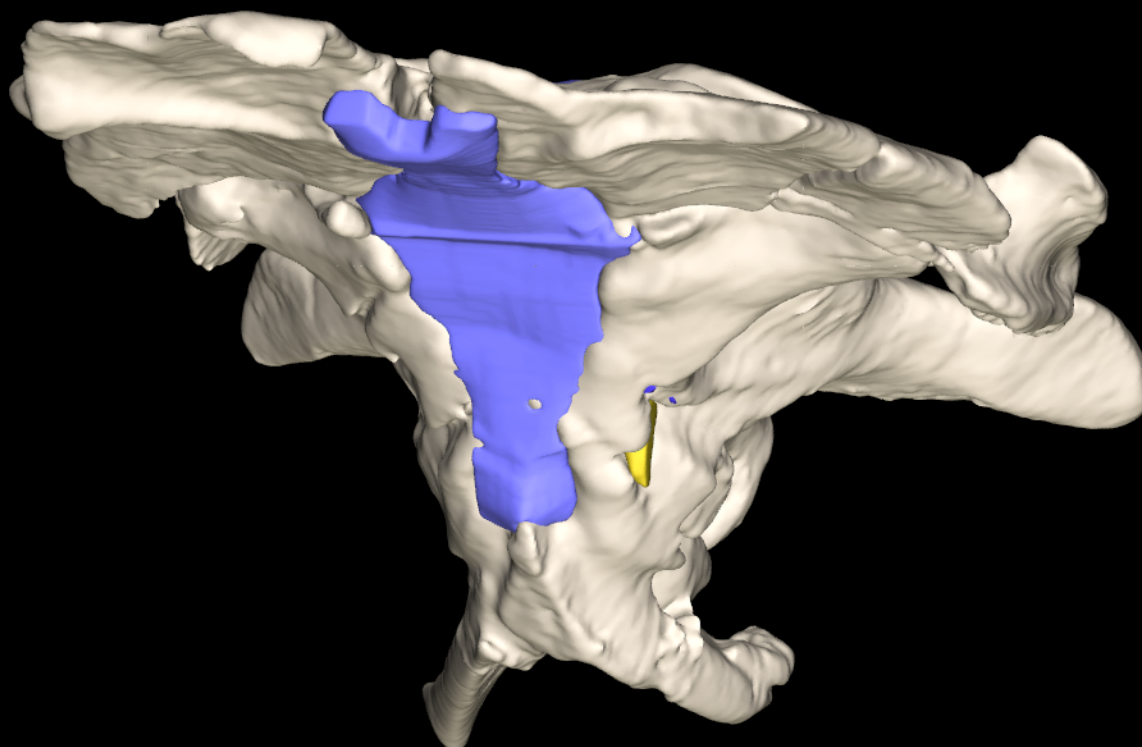

Supplement: Figure S2 — Interactive visualization made from the CT scan of the braincase of the sauropod dinosaur Spinophorosaurus nigerensis (GCP-CV-4229) from the Jurassic of Aderbissinat, Niger (medium file). (PDF) [file pone.0030060.s002.pdf]

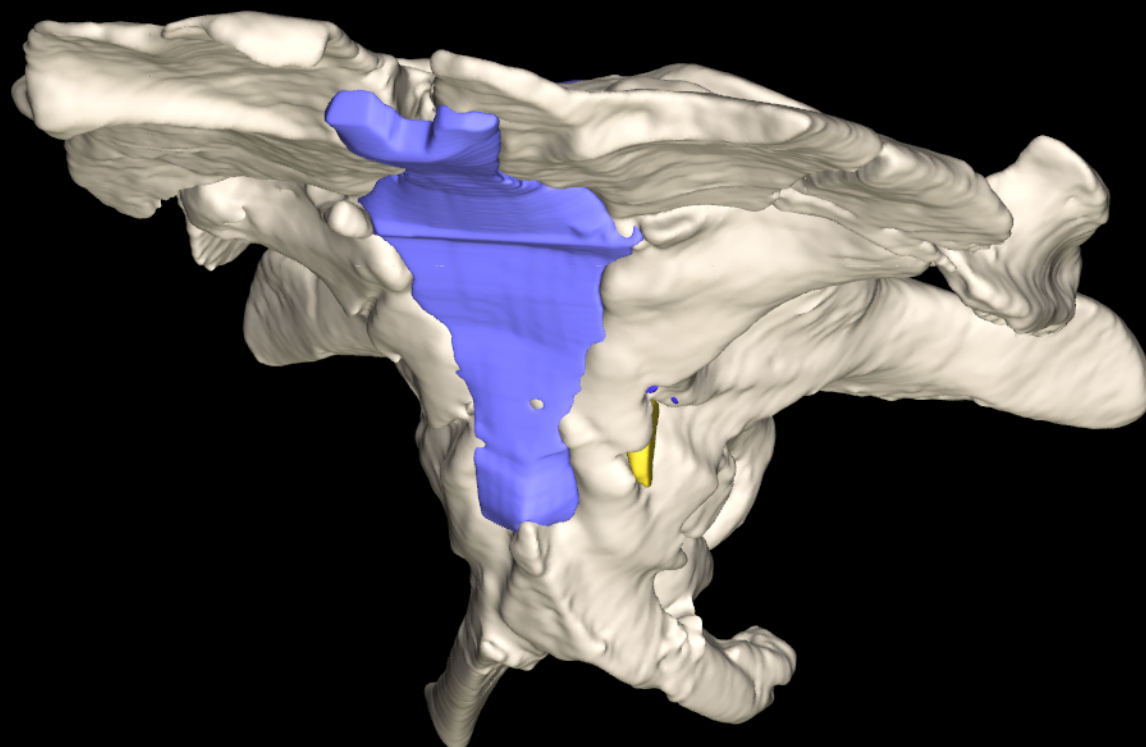

Supplement: Figure S3 — Interactive visualization made from the CT scan of the braincase of the sauropod dinosaur Spinophorosaurus nigerensis (GCP-CV-4229) from the Jurassic of Aderbissinat, Niger (large file). (PDF) [file pone.0030060.s003.pdf]
